# Supplementary figures and images for: Single-cell immune profiling of third trimester pregnancies defines importance of chemokine receptors and prevalence of CMV-induced NK cells in the periphery and decidua
Source: medRxiv. 2025 Mar 25:2025.03.24.25324489. Preprint. [Version 1] doi: 10.1101/2025.03.24.25324489 (PMC11974773; doi:10.1101/2025.03.24.25324489)

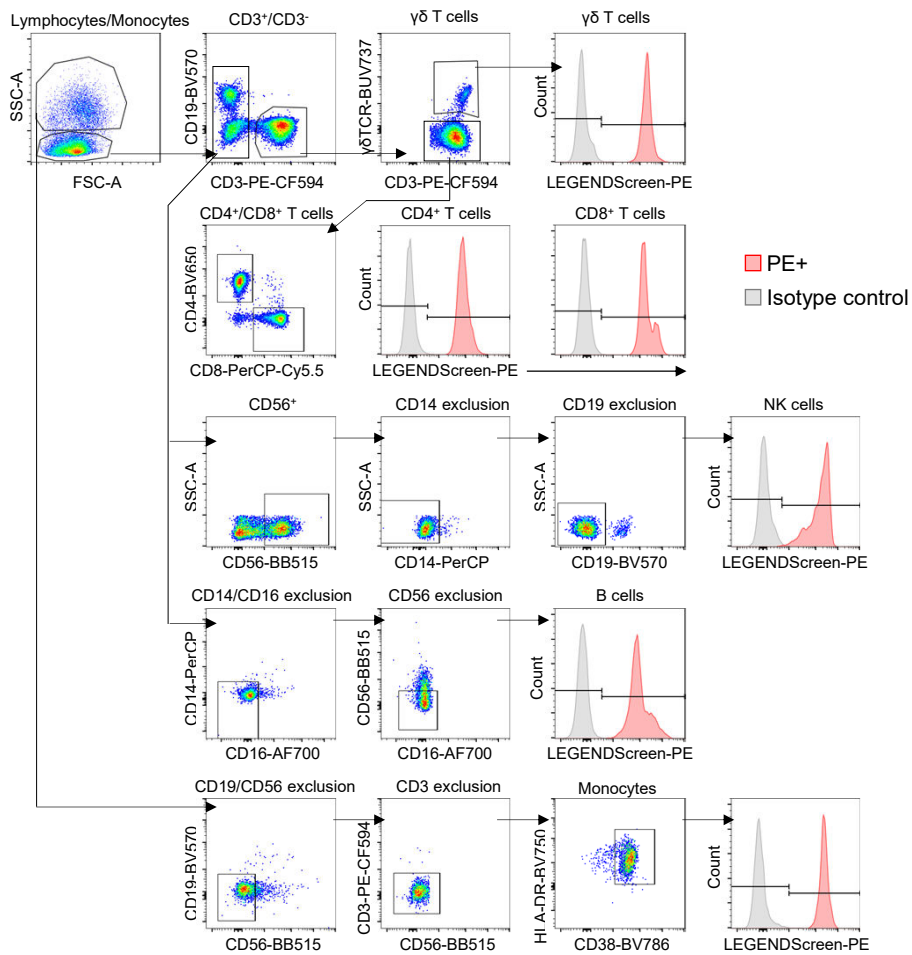

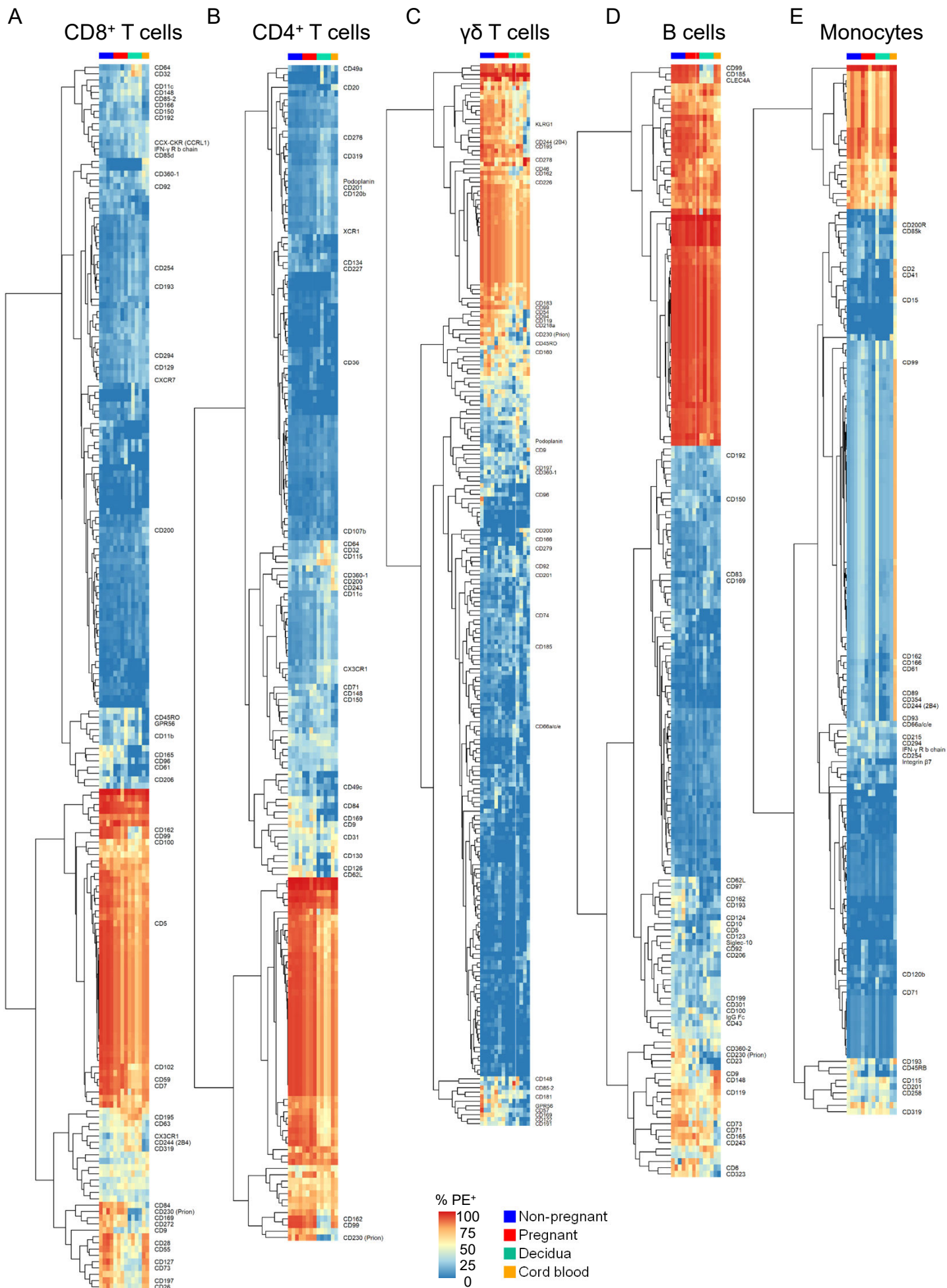

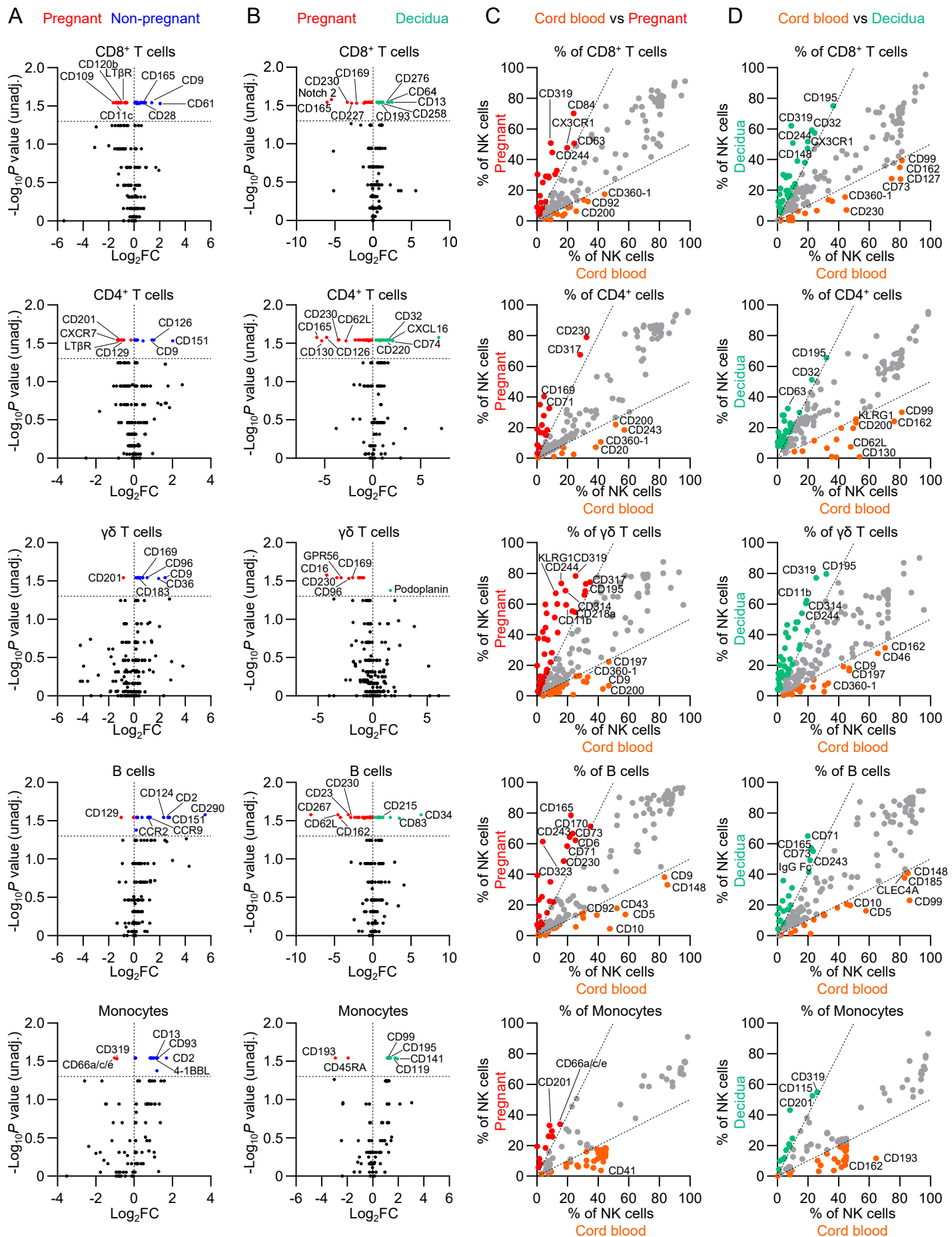

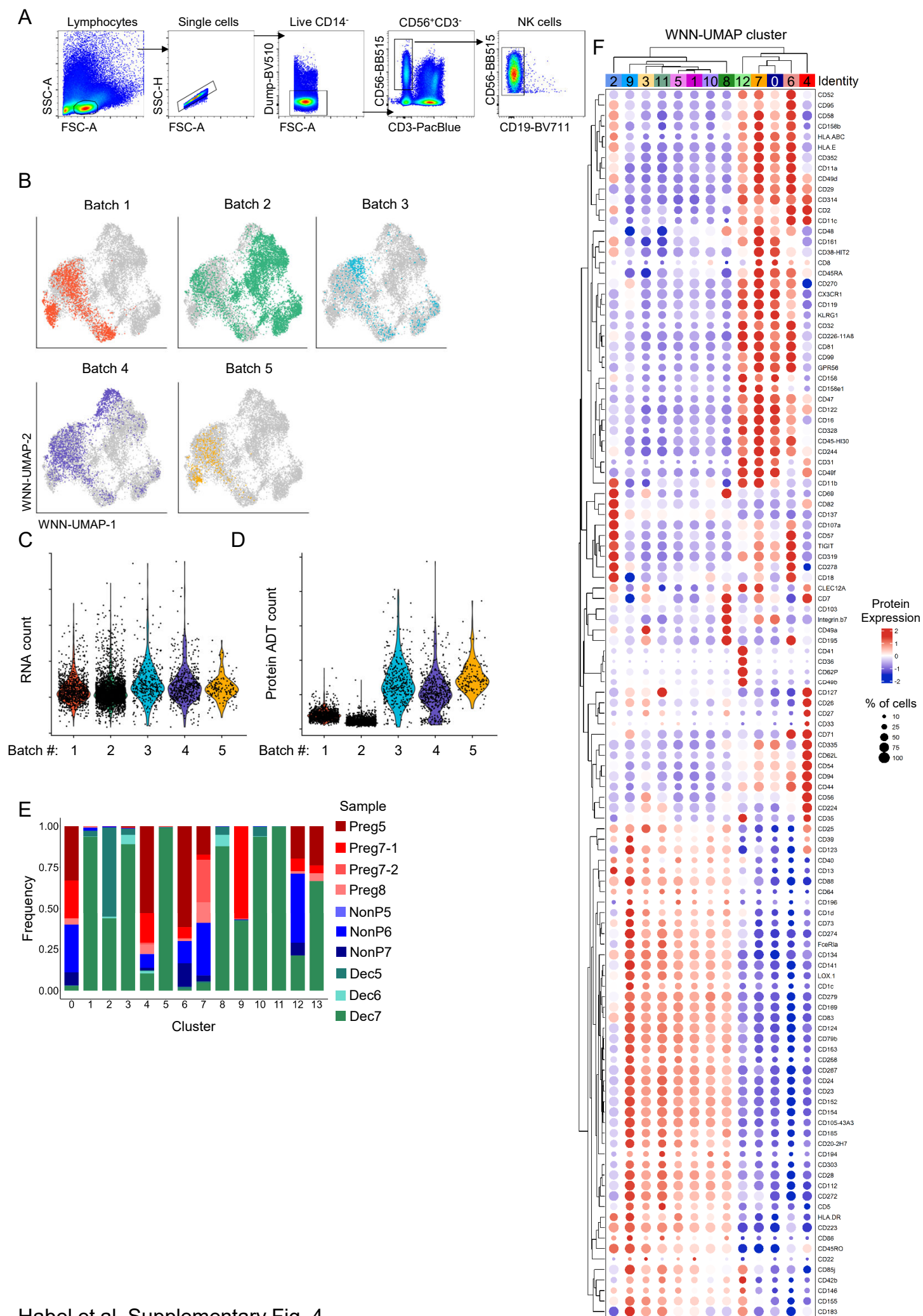

# NK development stages

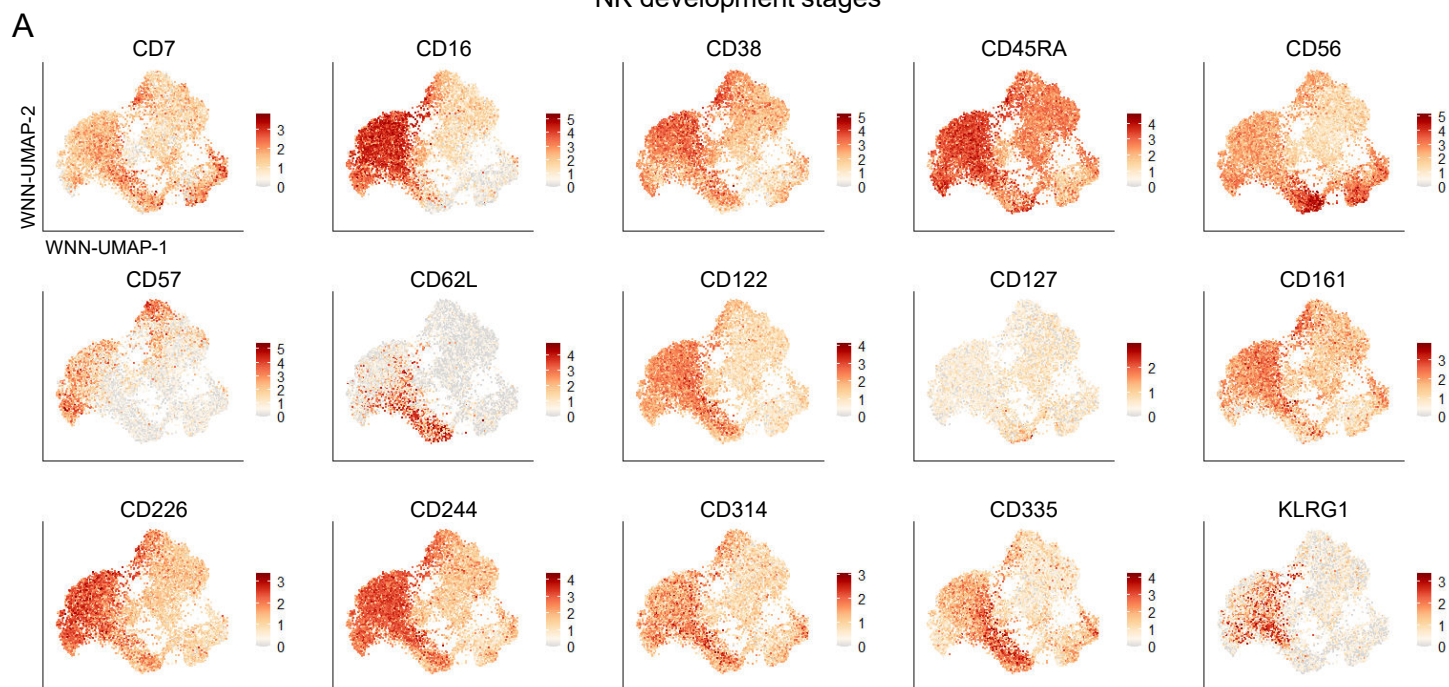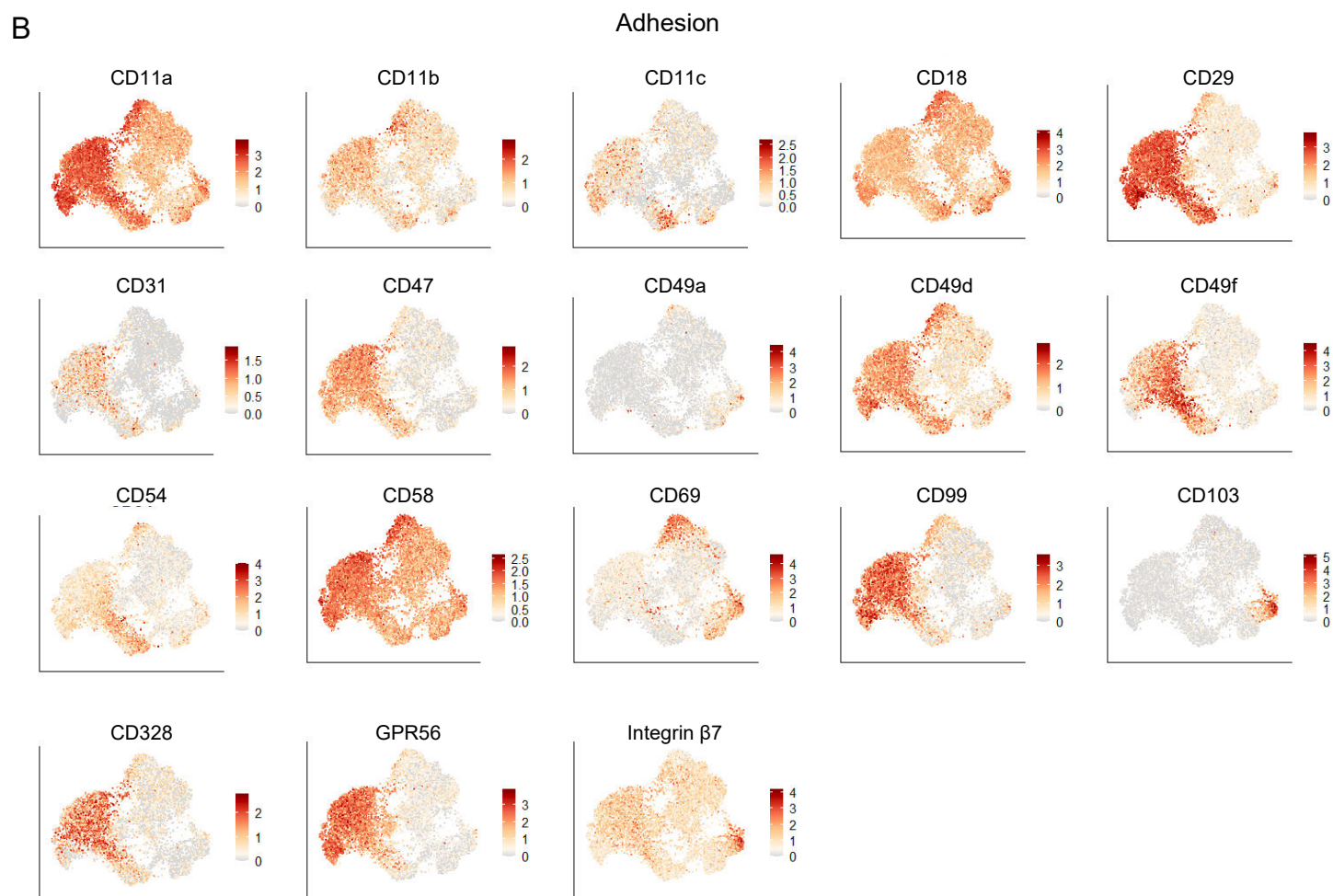

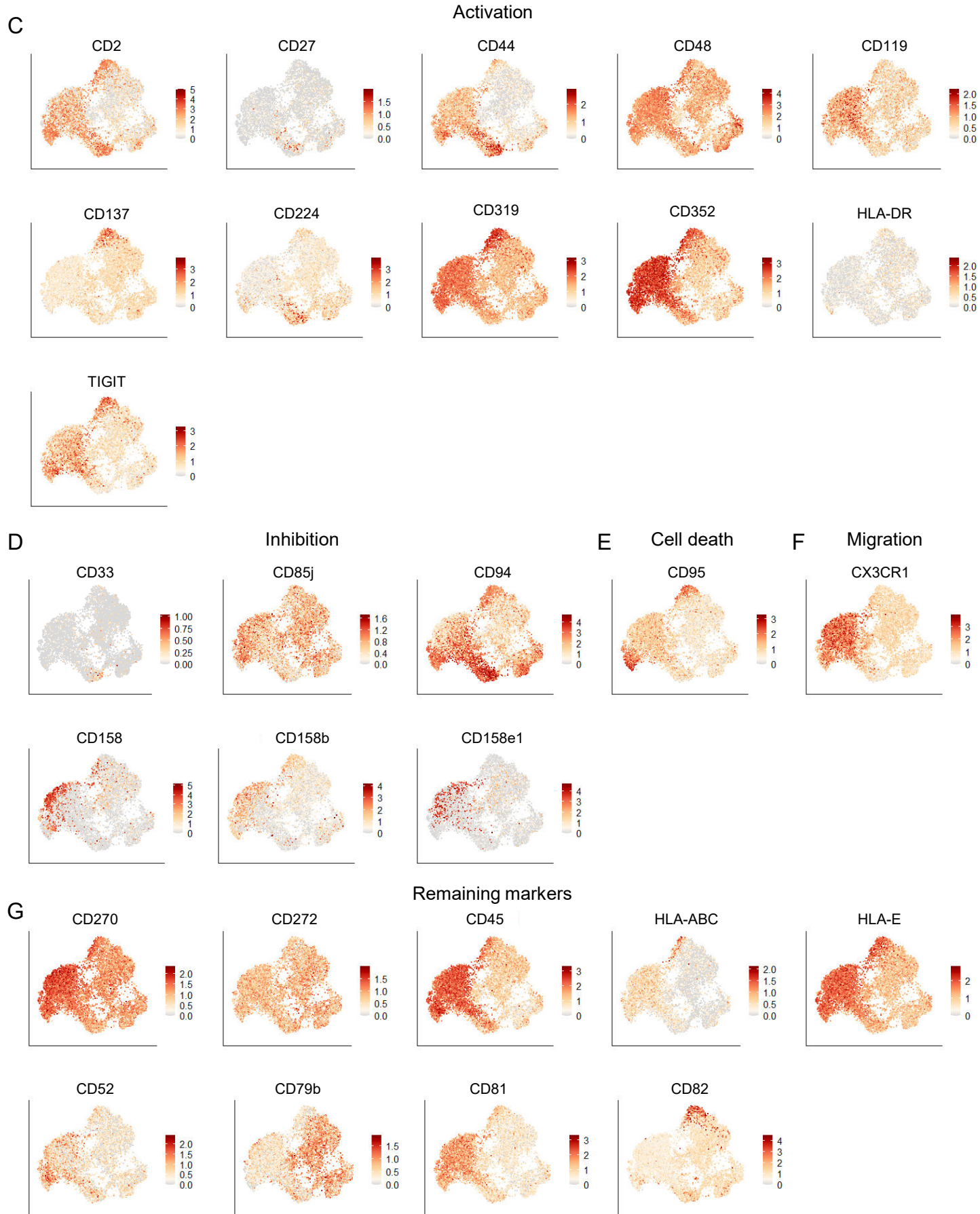

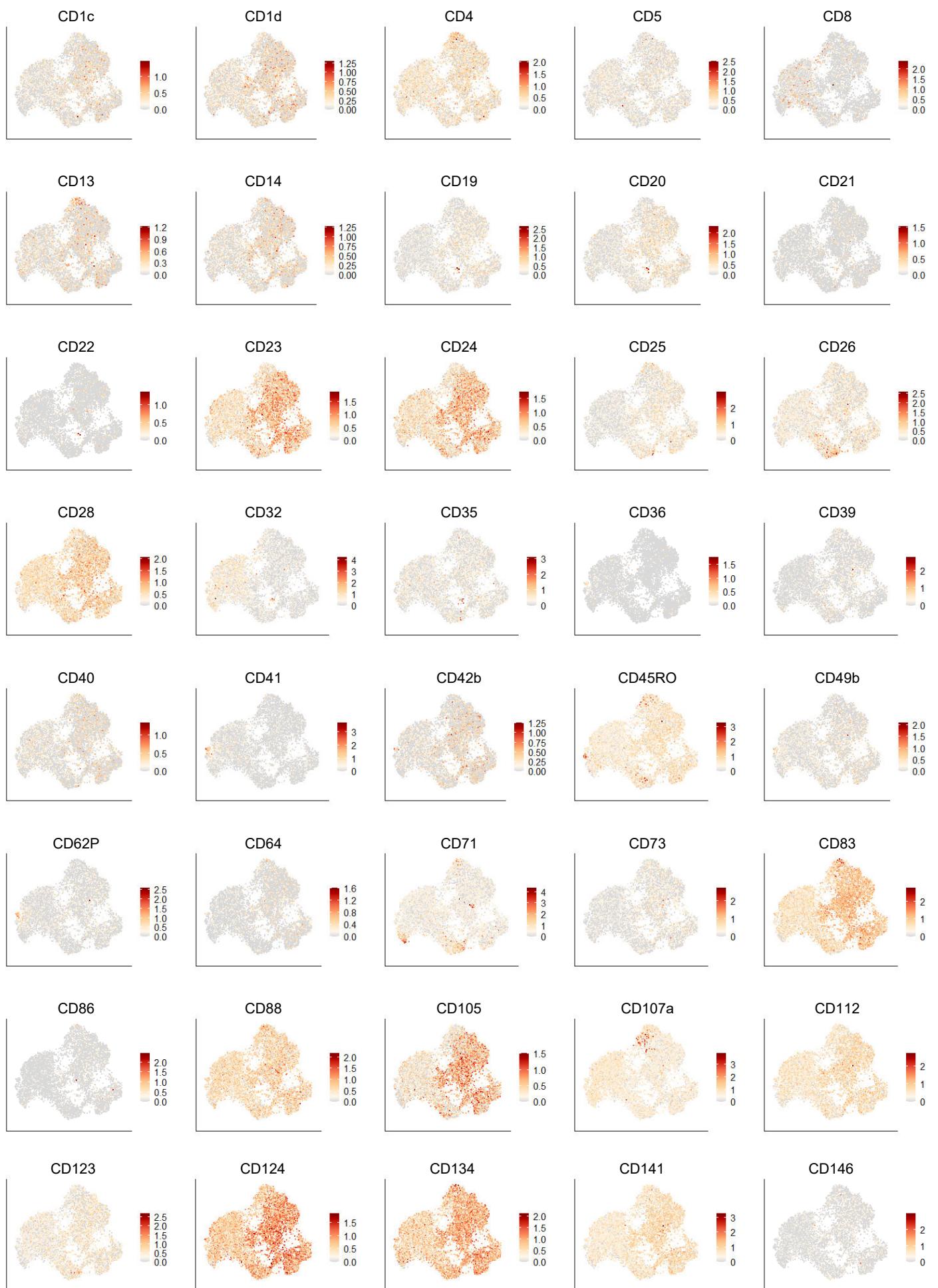

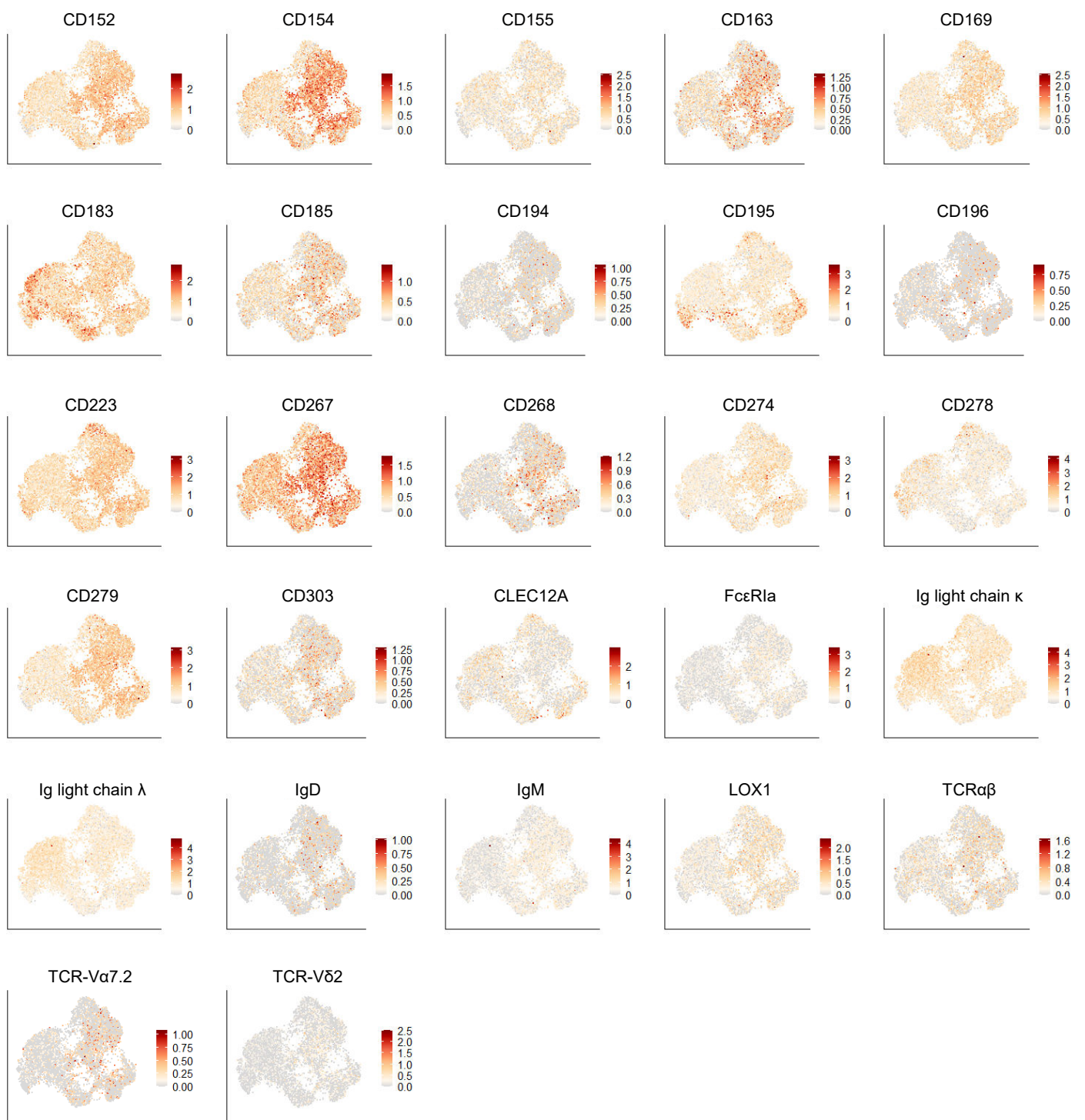

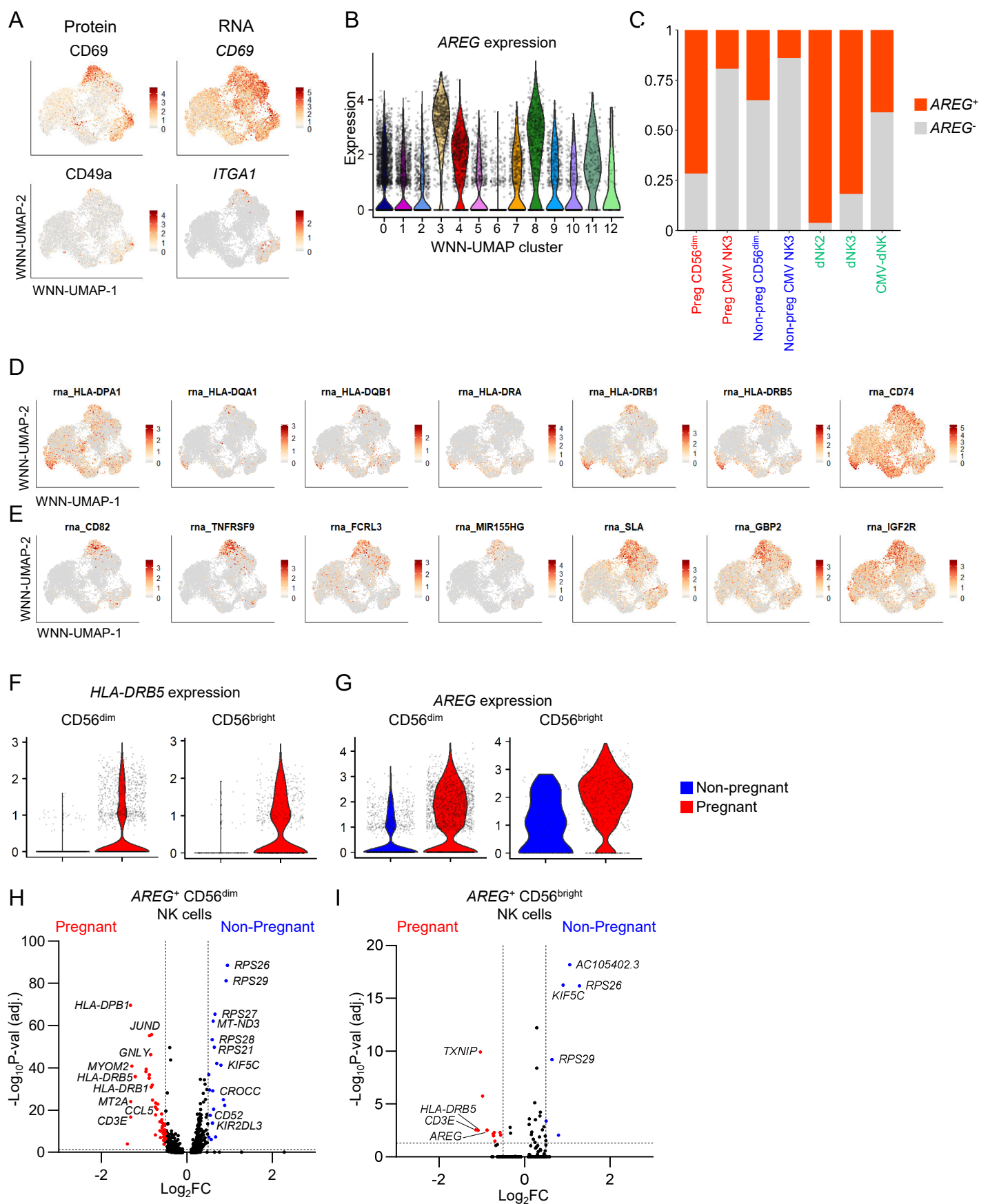



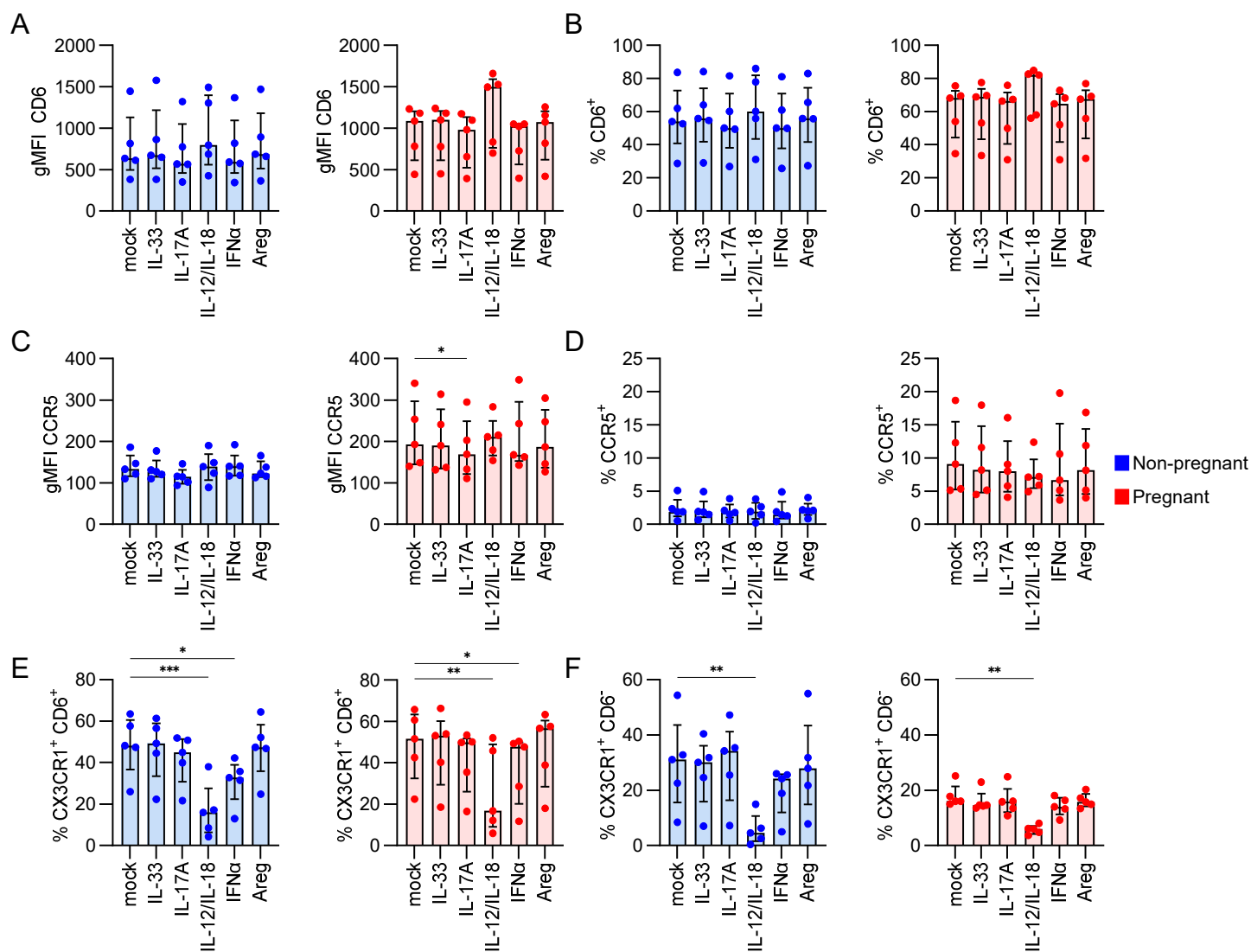

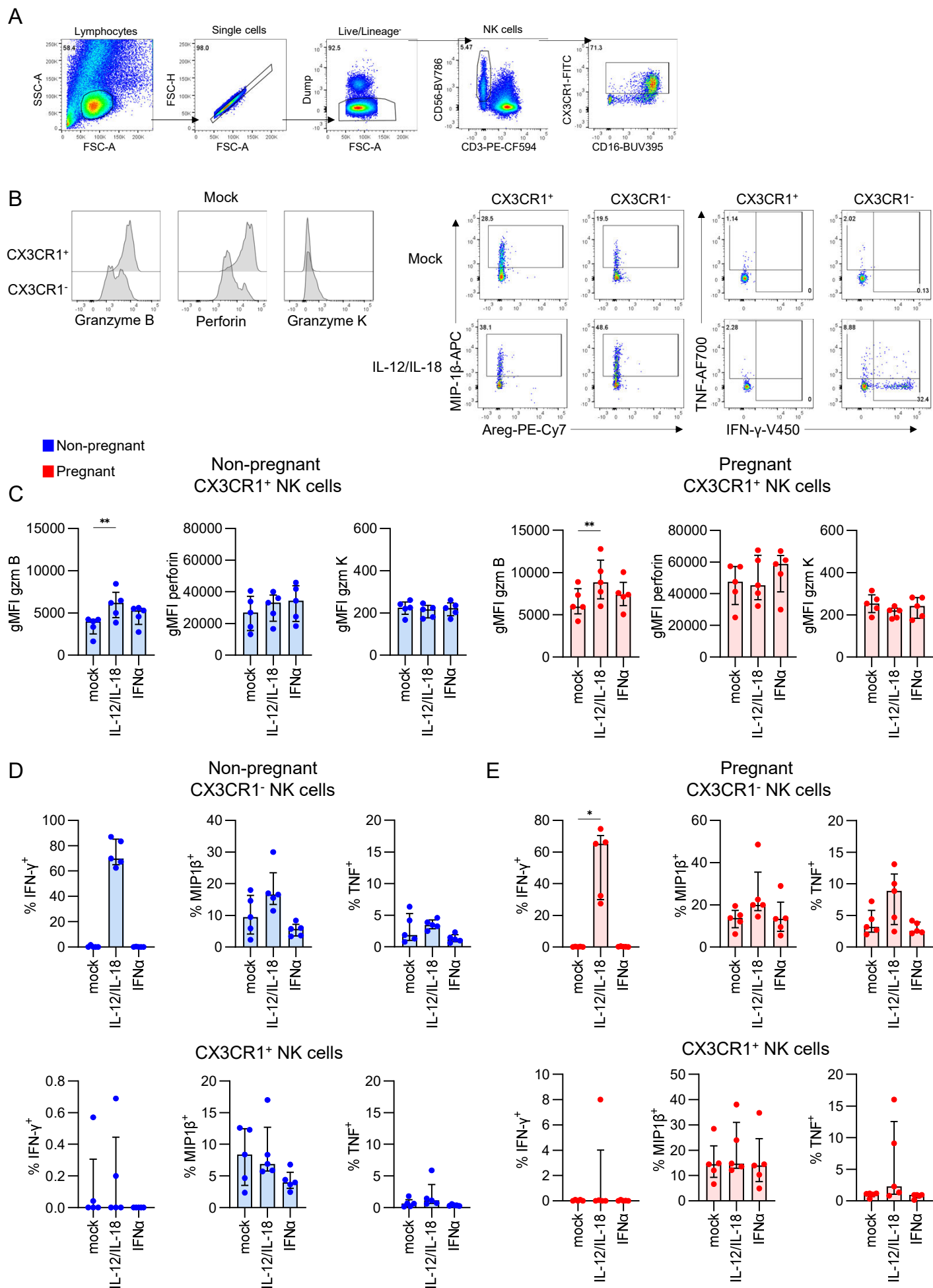

Supplement: Supplement 1 — Supplementary Figure 1. Gating strategy to identify NK cells, monocytes, B cells, CD8+ T cells, CD4+ T cells, and γδ T cells. Gating of LEGENDScreen mAb-PE signals (red) on histograms was based on respective isotype control (grey). Supplementary Figure 2. (A-E) Heatmaps with hierarchical clustering of columns showing frequencies of marker-PE expression on CD8+ T cells (A), CD4+ T cells (B), γδ T cells (C), B cells (D), and monocytes (E) from pregnant and non-pregnant peripheral blood, cord blood and decidua. Markers expressed on >10% of cells in at least one sample were included for analysis (196 (A), 188 (B), 238 (C), 178 (D), and 168 (E) out of total 342 markers). Supplementary Figure 3. (A) Volcano plots depicting differential protein expression in non-pregnant vs pregnant peripheral blood immune cell populations including, CD8+ T cells, CD4+ T cells, γδ T cells, B cells and monocytes. (B) Volcano plots depicting differential protein expression in decidual vs pregnant blood immune cell populations. Volcano plot horizontal dashed line indicates the log10 p-value cutoff of 1.3 (p=0.05). (C,D) Scatter plot of average percent of cells expressing for (C) cord blood vs pregnant peripheral blood and (D) cord blood vs decidual CD8+ T cells, CD4+ T cells, γδ T cells, B cells, monocytes. Scatter plot dashed lines indicate a log2FC cut-off of 1. Supplementary Figure 4. Sort gating strategy and quality control analysis of scRNAseq. (A) Gating strategy for bulk NK cells. (B) WNN-UMAP coloured by 10x reaction batches. (C) RNA and (D) antibody-derived-tag (ADT; C) read counts per cell, per batch of 10x reactions.(E) Proportion of each sample (pregnant peripheral blood NK cells (reds), non-pregnant peripheral blood NK cells (blues), decidua NK cells (teals) per WNN-UMAP cluster. (F) Hierarchically clustered dot plot of CITE-seq markers across each WNN-UMAP cluster. Supplementary Figure 5. WNN-UMAPs depicting each of the 130 surface proteins detected with CITE-seq oligo-labelled [file NIHPP2025.03.24.25324489v1-supplement-1.pdf]
